# Comparison of integral doses to normal tissue and organs at risk between interstitial high-dose-rate brachytherapy and modern external-beam radiotherapy techniques in breast and head and neck cancer patients

**DOI:** 10.1007/s00066-025-02382-3

**Published:** 2025-03-17

**Authors:** Tibor Major, Csaba Polgár, Zoltán Takácsi-Nagy

**Affiliations:** 1 Radiotherapy Centre, National Institute of Oncology, 7–9. Rath Gy. u., 1122 Budapest, Hungary; 2 Department of Oncology, Semmelweis University, Budapest, Hungary; 3 National Institute of Oncology and National Tumor Biology Laboratory, Budapest, Hungary

**Keywords:** Integral dose, Brachytherapy, External beam therapy, Breast cancer, Head and neck cancer

## Abstract

**Background:**

Although a cornerstone of modern cancer treatment, radiotherapy (RT) is associated with a risk of secondary cancer due to irradiation of non-target tissues. Techniques such as intensity-modulated RT (IMRT), volumetric modulated RT (VMAT), and stereotactic body RT (SBRT) provide highly conformal target dose distributions and reduce doses to nearby organs at risk (OARs), albeit at the cost of larger normal tissue volumes being irradiated with lower doses. In brachytherapy (BT), the low-value isodoses cannot be changed: they are a consequence of the inverse-square law. This study evaluates and compares the normal tissue integral doses (NTIDs) delivered by BT and modern external-beam RT (EBRT) techniques in breast and head and neck (H&N) cancer patients.

**Methods:**

Included were the BT and IMRT plans of 34 women with early-stage breast cancer treated with interstitial high-dose-rate (HDR) BT and two groups of head and neck (H&N) patients: 38 patients with mobile tongue, floor of the mouth, and base of the tongue cancer treated definitively with interstitial HDR BT for whom VMAT treatment plans were also prepared and 20 patients with tongue and floor of the mouth tumors who received postoperative interstitial HDR BT for whom VMAT and stereotactic CyberKnife (CK, Accuray Inc., Sunnyvale, CA, USA) plans were also created. The NTIDs for three normal tissue volumes (NT_V10, NT_V5, NT_V2) and OARs were calculated and compared.

**Results:**

Brachytherapy resulted in 39%, 32%, and 26% lower NTIDs compared to IMRT for NT_V10, NT_V5, and NT_V2, respectively, in patients with breast cancer. In H&N cancer, the NTIDs were always lower for BT compared to VMAT. The reductions in NTID achieved with BT were 45%, 36% and 27% with the same planning target volumes in BT and VMAT, and 56%, 48% and 37% with larger planning target volumes in VMAT. For CK, the NTID reductions were 54%, 49% and 41% compared to BT. In breast cases, BT resulted in a significant reduction in ipsilateral lung NTID, and in H&N cases, salivary glands NTIDs were always lower with BT than with VMAT.

**Conclusion:**

For patients with breast and head and neck cancer, interstitial BT results in lower integral dose to normal tissue and OARs compared to modern EBRT techniques. The clinical implications require further detailed investigation.

## Introduction

Radiotherapy is a safe and effective treatment modality for cancer patients that uses ionizing radiation to destroy malignant cells in the tumor. However, radiation can also induce secondary cancer in the body. Data show a possible association between the integral dose and a second cancer [1]. The integral dose represents the energy absorbed by the irradiated body. The normal tissue integral dose (NTID) is defined as the normal tissue volume multiplied by the mean dose. The normal tissue can be the whole body, a part of the body, or a body volume irradiated with a given dose. Normal tissue always contains only the non-target volume. The NTID can also be calculated for an organ at risk (OAR). The NTID is considered to be an important factor in the assessment of the risk of second primary malignancies [1–3].

Compared to traditional radiotherapy, modern radiotherapy techniques provide a highly conformal dose distribution to the target volume and reduce the dose to nearby OARs at the cost of larger normal tissue volumes being irradiated with lower doses. With the introduction of intensity-modulated radiotherapy (IMRT), volume modulated radiotherapy (VMAT), and stereotactic body radiotherapy (SBRT), the prescribed dose (PD) can be highly concentrated to the planning target volume (PTV), thus achieving excellent target dose coverage but lower doses distributed over a larger volume of the body. During treatment planning, the spatial distribution of low-dose volumes can be optimized by modifying beam arrangements and varying beam weights. In inverse planning, this is achieved by applying dose constraints to OARs and normal tissues. In brachytherapy (BT), the shape of low-value isodoses cannot be changed: they are nearly circular, and the volume they enclose depends on the distance from the source(s). This is a consequence of the inverse square law, which is the main factor shaping the dose distribution around the source.

The integral dose has been shown to be useful in assessing the incidence of second primary malignancies in patients treated by radiotherapy [1–3]. Several publications compare external-beam radiotherapy (EBRT) techniques in terms of the integral dose [2, 4–8]. However, comparative studies between EBRT and BT are very rare. Grzywacz et al. [9] performed a quantitative comparison between integral doses of high-dose-rate (HDR) BT and modern EBRT techniques for prostate cancer. Swetha et al. [10] also considered the integral dose in a dosimetric comparison between HDR BT and IMRT for cervical cancer. The integral dose can also be used to compare the dose distributions of different HDR BT sources and to characterize the dosimetry of external beams [11–13].

The objective of this study is to evaluate and compare the integral dose delivered by brachytherapy and modern external-beam radiotherapy techniques to normal tissue and organs at risk in breast and head and neck cancer patients.

## Materials and methods

### Patients and treatments

We selected treatment plans of patients who had previously been treated with irradiation to two tumor sites (breast and head and neck) at our institution. Thirty-four women with early-stage breast cancer were treated with multicatheter interstitial HDR BT using an Ir-192 source. In a previous study, the BT treatment plans were compared with the IMRT plans for organs at risk dosimetry [14]. In the comparative treatment plans, the same CT data and contours were used, with the same dose prescription of 7 × 4.3 Gy, in line with our clinical practice. In the BT plans, the planning target volume (PTV) was equal to the clinical target volume (CTV), while in the IMRT plans, the PTV was generated from the CTV with an isotropic margin of 5 mm.

The head and neck (H&N) patients included two groups. In the first group, 38 patients with mobile tongue, floor of the mouth, and base of the tongue cancer were treated definitively with interstitial HDR BT. In addition to the BT plans, VMAT treatment plans were also prepared using the same CT data and organ contours [15]. The second group included 20 patients with tongue and floor of the mouth tumors who received postoperative interstitial HDR BT [16]. VMAT and stereotactic CyberKnife (CK, Accuray Inc., Sunnyvale, CA, USA) plans were created for these patients in addition to BT plans. In clinical practice, different fractionation schemes are used for BT and EBRT. In order to make the comparison more realistic, the biologically effective dose (BED) was calculated for each treatment method using an α/β value of 3 Gy. A dose prescription of 15 × 3 Gy (BED_3_ = 90 Gy) was used in BT plans, 35 × 2 Gy (BED_3_ = 116.67 Gy) in 38 H&N definitive plans, and 30 × 2 Gy (BED_3_ = 100 Gy) in 20 postoperative H&N plans. With SBRT on CyberKnife, the schedule was 5 × 7 Gy (BED_3_ = 116.67 Gy). The subscript “3” in BED_3_ refers to α/β = 3 Gy. In the VMAT plans a 3-mm margin and in the CK plans a 2-mm margin was added to the CTV to create the PTV.

For BT the Oncentra Brachy v4.3 (Elekta, Brachytherapy, Veenendaal, the Netherlands), for IMRT the Eclipse v11 (Varian Medical Systems, Palo Alto, CA, USA), and for CK the Precision 2.0.0.1 (Accuray Inc., Sunnyvale, CA, USA) planning systems were used.

### Integral doses

**Fig. 1:**
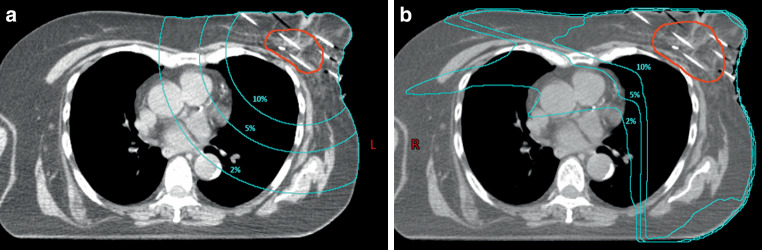
Volumes of normal tissue irradiated by 10% (NT_V10), 5% (NT_V5) and 2% (NT_V2) of the prescribed dose for **a** brachytherapy and **b** intensity modulated radiotherapy for a breast cancer patient

**Fig. 2:**
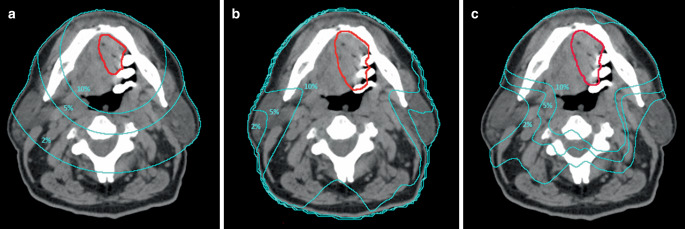
Volumes of normal tissue irradiated by 10% (NT_V10), 5% (NT_V5), and 2 % (NT_V2) of the prescribed dose for **a** brachytherapy, **b** volume modulated radiotherapy and **c** stereotactic CyberKnife radiotherapy for a head and neck cancer patient

The NTIDs for three normal tissue volumes and organs at risk were calculated in the current study. We first created normal tissue structures from the volumes surrounded by the 10%, 5%, and 2% isodose surfaces, and then subtracted the volume of the PTV from them to create the volumes of NT_V10, NT_V5, and NT_V2, respectively. Figure 1 shows the three volumes on a transverse CT slice of a breast cancer patient. The same volumes of a H&N cancer patient are presented in Fig. 2. In the planning systems, the average dose for each generated volume was calculated and then multiplied by the volume to obtain the NTID value. In the literature, Gy · liter is the most commonly used unit of measurement for NTID, but joule (J) is also applied [6, 7, 12, 17–20]. The volumes can be measured in cubic centimeters, doses can be expressed as a percentage of the PD (relative dose), and the NTID can be calculated by multiplying these, allowing for a direct comparison when the dose prescription varies between different treatment modalities [9]. Another way to compare the NTID between treatment schedules with a different dose per fraction is to use the BED instead of the physical dose [5]. In the current study, BED_3_ was calculated for each treatment method given in Gy_2_, where the subscript “2” represents the reference dose of 2 Gy per fraction, and the unit of the NTID is Gy_2_ · liter [5]. In addition to normal tissues, the integral doses to organs at risk were also calculated and compared. For breast cases, the ipsilateral lung and the ipsilateral non-target breast were evaluated; for H&N cases, the salivary glands (bilateral parotid and submandibular glands) were evaluated.

### Statistical analysis

Descriptive statistics were used to characterize the volumes, mean doses, and integral doses. The Shapiro–Wilk test was used to check the normality of the data distribution. As most of the parameters were not normally distributed, the Wilcoxon matched-pairs signed-rank test was used for all comparisons. The difference was considered significant if the *p*-value was <0.05. Statistical evaluation was performed using Prism 8.0.1 software (GraphPad Software Inc., Boston, MA, USA).

## Results

**Fig. 3:**
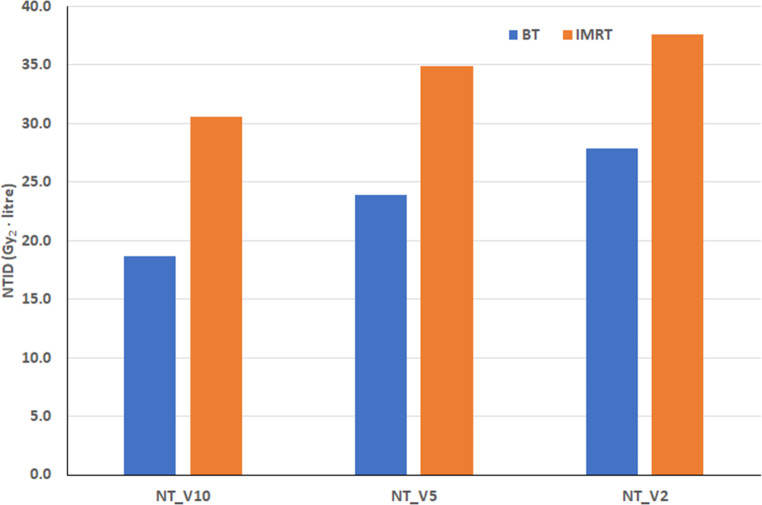
Mean normal tissue integral doses (NTID) of brachytherapy (BT) and intensity modulated radiotherapy (IMRT) for breast cancer patients. Gy_2_ means biologically effective dose with a 2-Gy fractional dose, and NT_VX indicates the volume of normal tissue irradiated by X% of the prescribed dose

For breast patients, the difference between BT and IMRT in normal tissue volumes irradiated by low doses depends on the dose value (Table 1). For the region receiving 10% of the PD, the volume for BT is 30% lower, while for the region receiving 2% of the PD, it is 27% higher compared to the IMRT. However, for the mean doses, the difference increases as the dose decreases (Table 1). For NT_V10, the mean dose of BT is 14% lower compared to IMRT, and this difference reaches 43% for NT_V2. Consequently, the integral doses for the three normal tissue volumes are lower for BT than for IMRT (Fig. 3). Brachytherapy resulted in 39%, 32%, and 26% lower NTIDs for NT_V10, NT_V5, and NT_V2, respectively. The difference was statistically significant for all volumes. The figure also shows that the integral dose of the normal tissue volume increases as the dose decreases: NTDI for NT_V2 of BT is 49% higher than for NT_V10. For IMRT, the increase is 23%.

**Table 1:** Mean volumes and mean doses (with ranges) of normal tissue irradiated by relative doses for brachytherapy (BT) and intensity-modulated radiotherapy (IMRT) of 34 breast patients

	BT	IMRT	BT/IMRT
*Volume (cm*^*3*^*)*			
NT_V10	971.7 (546.9–1928.5)	1393.9 (808.3–2368.7)	0.70
NT_V5	1975.6 (1174.3–3478.7)	2190.9 (1196.4–4190.1)	0.90
NT_V2	4036.4 (1080.6–6604.8)	3178.3 (1683.6–5595.1)	1.27
PTV	60.4 (26.9–173.6)	124.6 (67.3–311.3)	0.48
*Dose (Gy*_*2*_*)*			
NT_V10	19.0 (15.5–22.0)	22.1 (17.1–27.0)	0.86
NT_V5	11.9 (9.4–14.5)	16.1 (13.1–21.0)	0.74
NT_V2	6.8 (3.7–9.2)	11.9 (10.0–16.3)	0.57

*NT_VX* volume of normal tissue irradiated by X% of the prescribed dose, *Gy*_*2*_ biologically effective dose with a 2-Gy fractional dose

**Fig. 4:**
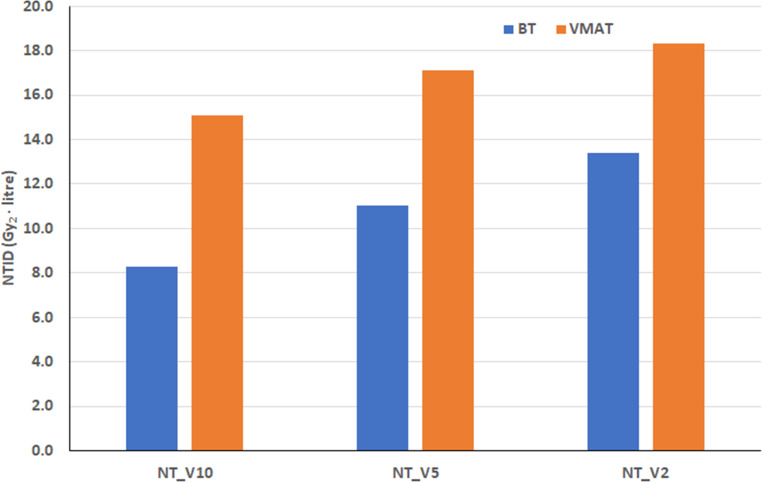
Mean normal tissue integral doses (NTID) of brachytherapy (BT) and volume modulated radiotherapy (VMAT) for head and neck patients. The volume of the PTV was the same in BT and VMAT plans. Gy_2_ means biologically effective dose with a 2-Gy fractional dose, and NT_VX indicates the volume of normal tissue irradiated by X% of the prescribed dose

**Fig. 5:**
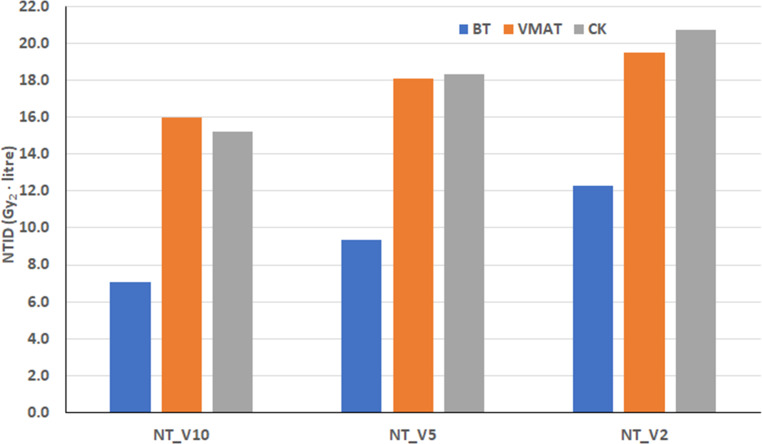
Mean normal tissue integral doses (NTID) of brachytherapy (BT), volume modulated radiotherapy (VMAT) and stereotactic CyberKnife (CK) radiotherapy for head and neck patients. The volume of the PTV in VMAT and CK plans was larger than in BT plans. Gy_2_ means biologically effective dose with a 2-Gy fractional dose, and NT_VX indicates the volume of normal tissue irradiated by X% of the prescribed dose

The characteristics of normal tissue volumes, mean doses, and integral doses observed in H&N patients were similar to those seen in breast patients (Tables 2 and 3; Figs. 4 and 5). The volume of 10% (NT_V10) is smaller and the volume of 2% (NT_V2) is larger for BT compared to EBRT (VMAT and CK; Tables 2 and 3). However, the difference in mean doses is always largest for NT_V2 and smallest for NT_V10 (Tables 2 and 3). The integral doses for the three normal tissue volumes are always lowest for BT (Figs. 4 and 5). In both H&N groups, the difference was statistically significant for all volumes when BT was compared to VMAT. The CK values were similar to VMAT (Fig. 5) and significantly higher than those for BT.

**Table 2:** Mean volumes and doses (with ranges) of normal tissue irradiated by relative doses for brachytherapy (BT) and volume modulated radiotherapy (VMAT) of 38 head and neck patients with definitive radiotherapy. The volumes of the planning target volumes for VMAT were the same as for BT

	BT	VMAT	BT/VMAT
*Volume (cm*^*3*^*)*			
NT_V10	390.2 (176.0–770.9)	460.6 (238.7–803.5)	0.85
NT_V5	819.5 (380.7–1493.3)	716.7 (429.2–1351.5)	1.14
NT_V2	1584.2 (750.5–2832.9)	1037.5 (548.4–1853.6)	1.53
PTV	16.5 (5.2–42.3)	16.7 (5.6–43.5)	0.99
*Dose (Gy*_*2*_*)*			
NT_V10	21.1 (19.4–23.1)	34.6 (31.6–38.4)	0.61
NT_V5	13.3 (11.2–16.2)	25.3 (21.7–33.1)	0.53
NT_V2	8.4 (5.1–14.8)	19.0 (13.7–27.1))	0.44

*NT_VX* volume of normal tissue irradiated by X% of the prescribed dose, *Gy*_*2*_ biologically effective dose with a 2-Gy fractional dose

**Table 3:** Mean volumes and doses (with ranges) of normal tissue irradiated by relative doses for brachytherapy (BT) and volume modulated radiotherapy (VMAT) of 20 head and neck patients with postoperative radiotherapy. The volumes of the planning target volumes for VMAT and CyberKnife (CK) were larger than for BT

	BT	VMAT	CK	BT/VMAT	BT/CK
*Volume (cm*^*3*^*)*					
NT_V10	321.9 (105.5–721.0)	562.2 (227.7–1071.9)	533.7 (241.9–867.6)	0.57	0.60
NT_V5	679.4 (263.2–1363.8)	862.5 (410.8–1531.3)	907.9 (407.0–1446.4)	0.79	0.75
NT_V2	1630.5 (683.7–2874.8)	1205.8 (586.7–2122.4)	1536.3 (673.4–2318.0)	1.35	1.06
PTV	12.5 (2.6–37.1)	26.5 (7.7–65.3)	20.5 (5.6–51.6)	0.47	0.61
*Dose (Gy*_*2*_*)*					
NT_V10	21.8 (20.7–23.0)	28.4 (26.6–32.2)	28.6 (24.6–33.1)	0.77	0.76
NT_V5	13.6 (11.8–15.2)	21.1 (17.9–25.3)	20.2 (17.2–22.9)	0.64	0.67
NT_V2	7.4 (6.3–8.7)	16.4 (12.9–20.5)	13.5 (11.0–15.6)	0.45	0.55

*NT_VX* volume of normal tissue irradiated by X% of the prescribed dose, *Gy*_*2*_ biologically effective dose with a 2-Gy fractional dose, CK CyberKnife (Accuray Inc., Sunnyvale, CA, USA)

The differences between the two groups of H&N patients are mainly due to the different PTVs and fractionation schemes. In the group of 38 patients, the PTV was always the same in the BT and VMAT plans, with a mean volume of 16.5 cm^3^ (range 5.2–42.3 cm^3^). However, in the group of 20 patients, the PTV volumes were larger in the VMAT plans due to the 3‑mm CTV–PTV safety margin (26.5 cm^3^ vs. 12.5 cm^3^). The ranges are 7.7–65.3 cm^3^ and 2.6–37.1 cm^3^ for VMAT and BT, respectively. For the CK, the mean volume of the PTV was 20.5 cm^3^ (range 5.6–51.6 cm^3^).

With the same treatment technique and dose prescription, the NTID correlates with the size of the PTV. In the H&N patients treated with BT, the NTIDs were smaller in the group of 20 patients because the volumes of the PTVs were smaller (mean 12.5 cm^3^ vs. 16.5 cm^3^). Patients in the 20-patient group treated with VMAT had larger PTVs (26.5 cm^3^ vs. 16.7 cm^3^) and BED_3_ values (116.67 Gy_2_ vs. 100 Gy_2_) compared to the 38-patient group, thus resulting in different NTID values.

Table 4 shows the volumes, mean doses, and NTIDs of the ipsilateral lung and ipsilateral non-target breast as organs at risk in the breast cancer patients. For BT, the mean dose of the ipsilateral lung was 33% less than for IMRT (*p* < 0.05). The mean dose of the ipsilateral non-target breast was also lower for BT, by 7% (*p* = 0.09). There was also a statistically significant difference in NTID in favor of BT for the ipsilateral lung (3.92 Gy_2_ · liter vs. 5.89 Gy_2_ · liter). For the ipsilateral non-target breast, NTID was 12.87 Gy_2_ · liter for BT and 13.29 Gy_2_ · liter for IMRT (*p* = 0.5542).

**Table 4:** Mean volumes and mean doses (with ranges) of organs at risk for brachytherapy (BT) and intensity-modulated radiotherapy (IMRT) of 34 breast patients

	BT	IMRT	BT/IMRT
*Volume (cm*^*3*^*)*			
Ipsilateral lung	1100.6 (622.5–1952.1)	1100.6 (622.5–1952.1)	1.00
Ipsilateral non-target breast	840.1 (276.9–2040.5)	770.5 (195.2–2021.3)	1.09
*Mean dose (Gy*_*2*_*)*			
Ipsilateral lung	3.59 (1.46–5.64)	5.35 (0.59–9.45)	0.67
Ipsilateral non-target breast	17.80 (6.79–36.84)	19.04 (7.98–29.66)	0.93
*Integral dose (Gy*_*2*_* · liter)*			
Ipsilateral lung	3.92 (1.51–7.25)	5.89 (0.48–14.45)	0.67^a^
Ipsilateral non-target breast	12.87 (5.81–25.10)	13.29 (5.36–26.79)	0.97

*Gy*_*2*_ biologically effective dose with a 2-Gy fractional dose

^a^Difference is statistically significant

**Fig. 6:**
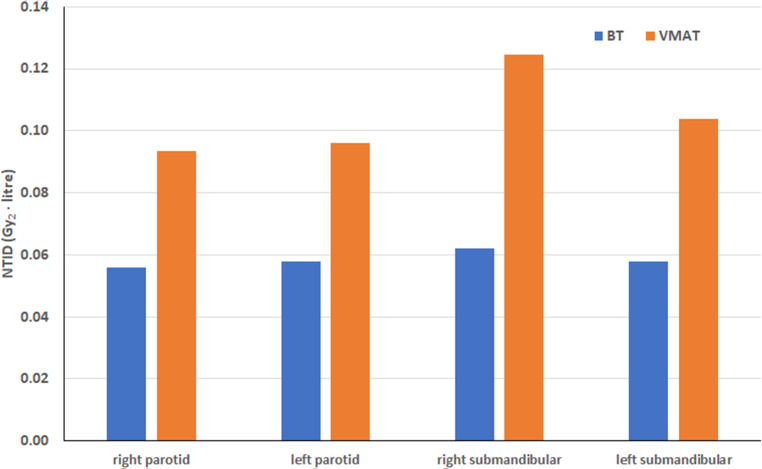
Mean organs at risk integral doses (NTID) of brachytherapy (BT) and volume modulated radiotherapy (VMAT) for head and neck patients. The volume of the PTV was the same in BT and VMAT plans. Gy_2_ means biologically effective dose with a 2-Gy fractional dose.

For H&N patients, the integral doses to the salivary glands were compared. Table 5 shows the volumes and doses. The NTID for the bilateral parotid and submandibular glands is shown graphically in Fig. 6. In this comparison, the PTV volumes were the same for BT and VMAT. The mean dose to each gland was significantly lower for BT than for VMAT, resulting in a significantly lower integral dose for BT. An approximately 40% reduction was observed in both parotid glands and a 51% reduction in the right and a 46% reduction in the left submandibular glands. For postoperative H&N cases, volume and dose data are given in Table 6. The mean doses in the glands are lowest for CK, resulting in the lowest integral doses. BT resulted in lower doses to the parotids and submandibular glands compared to VMAT; therefore, the integral dose in each gland was also lower with BT, with a reduction between 4% and 27%. The difference was significant only for the right parotid gland (*p* = 0.04).

**Table 5:** Mean volumes and mean doses (with ranges) of salivary glands for brachytherapy (BT) and volume modulated radiotherapy (VMAT) of 38 head and neck patients. The volumes of planning target volumes for VMAT were the same as for BT

	BT	VMAT	BT/VMAT
*Volume (cm*^*3*^*)*			
Right parotid	17.0 (3.1–42.4)	16.7 (2.5–42.9)	1.02
Left parotid	18.4 (3.9–45.5)	18.6 (4.0–45.8)	0.99
Right submandibular	6.6 (0.4–11.1)	6.7 (0.5–11.5)	0.99
Left submandibular	6.8 (0.4–13.9)	6.9 (0.5–14.2)	0.99
*Dose (Gy*_*2*_*)*			
Right parotid	3.4 (1.2–6.7)	4.9 (1.1–12.2)	0.69
Left parotid	3.2 (1.3–5.6)	4.8 (0.9–10.6)	0.67
Right submandibular	11.3 (2.8–37.2)	23.9 (0.8–81.3)	0.47
Left submandibular	9.2 (3.0–26.6)	19.3 (0.8–63.5)	0.48
*Integral dose (Gy*_*2*_* · liter)*			
Right parotid	0.056 (0.006–0.183)	0.093 (0.004–0.484)	0.60^a^
Left parotid	0.060 (0.014–0.160)	0.096 (0.005–0.289)	0.62^a^
Right submandibular	0.061 (0.009–0.245)	0.124 (0.004–0.230)	0.49^a^
Left submandibular	0.057 (0.006–0.237)	0.105 (0.004–0.230)	0.54^a^

*Gy*_*2*_ biologically effective dose with a 2-Gy fractional dose

^a^Difference is statistically significant

**Table 6:** Mean volumes and doses (with ranges) of salivary glands for brachytherapy (BT), volume modulated radiotherapy (VMAT) and CyberKnife (CK) radiotherapy of 20 head and neck patients. The volumes of PTV for VMAT and CK were larger than for BT

	BT	VMAT	CK	BT/VMAT	BT/CK
*Volume (cm*^*3*^*)*					
Right parotid	24.0 (3.6–42.0)	23.3 (3.8–42.4)	23.7 (4.0–43.0)	1.03	1.01
Left parotid	23.2 (1.3–44.9)	22.9 (2.7–44.3)	23.3 (3.0–45.0)	1.01	1.00
Right submandibular	8.5 (1.9–12.3)	8.6 (2.1–12.6)	8.8 (2.2–12.8)	0.98	0.97
Left submandibular	8.1 (2.4–13.1)	8.2 (2.6–13.3)	8.4 (2.7–13.6)	0.99	0.96
*Mean dose (Gy*_*2*_*)*					
Right parotid	2.5 (1.–6.7)	3.7 (0.4–6.8)	1.1 (0.2–3.2)	0.68	2.27
Left parotid	2.5 (0.9–4.3)	3.0 (0.4–5.1)	1.0 (0.1–2.1)	0.83	2.50
Right submandibular	6.4 (2.3–13.5)	6.9 (2.5–14.9)	2.2 (1.0–3.7)	0.93	2.91
Left submandibular	6.1 (2.9–11.5)	6.9 (0.9–14.1)	2.3 (0.7–5.0)	0.88	2.65
*Integral dose (Gy*_*2*_* · liter)*					
Right parotid	0.060 (0.012–0.145)	0.082 (0.011–0.222)	0.026 (0.002–0.089)	0.73^a^	2.31^a^
Left parotid	0.060 (0.004–0.125)	0.069 (0.007–0.199)	0.023 (0.001–0.060)	0.87	2.61^a^
Right submandibular	0.047 (0.016–0.045)	0.053 (0.020–0.086)	0.018 (0.006–0.028)	0.89	2.61^a^
Left submandibular	0.053 (0.011–0.151)	0.055 (0.004–0.132)	0.023 (0.003–0.068)	0.96	2.30^a^

*Gy*_*2*_ biologically effective dose with a 2-Gy fractional dose

^a^Difference is statistically significant, CK CyberKnife (Accuray Inc., Sunnyvale, CA, USA)

## Discussion

Publications on integral dose comparisons include a relatively small number of patients. Shi et al. [5] compared the treatment plans of step-and-shoot IMRT and helical tomotherapy in 6 patients for six treatment sites (one case per treatment site). They found that, depending on the treatment site, tomotherapy can produce plans with lower integral doses to healthy organs compared to IMRT. Yang et al. [6] selected 10 patients with endometrial cancer who underwent postoperative whole-pelvic radiotherapy using 3D-CRT, IMRT, and helical tomotherapy. Their results show that tomotherapy provides lower integral doses to OARs, and integral doses to normal tissue and the whole body were lower with IMRT.

In another study, the integral doses of VMAT, helical tomotherapy, and 3D-CRT of craniospinal irradiation were compared in 5 children with medulloblastoma [8]. The mean non-target normal tissue integral dose was lowest with VMAT and highest with tomotherapy. Slosarek et al. [7] performed a comparison of integral doses between four EBRT techniques (helical tomotherapy, stereotactic CyberKnife, IMRT, and VMAT) for prostate radiotherapy using the treatment plans of 10 patients. They observed the highest total dose absorbed by normal tissue with tomotherapy and CK.

To our knowledge, this is the first study to directly compare NTIDs between interstitial brachytherapy and modern external-beam radiotherapy in breast and head and neck cancer patients. Grzywacz et al. [9] compared NTID for four radiotherapy techniques commonly used to treat prostate cancer. High-dose-rate BT, VMAT, SBRT, and proton therapy plans were prepared for 10 patients, and the integral dose to normal tissue structures (CT-scanned volume minus PTV) was calculated for each technique. NTID was lowest for BT, and the dose reduction achievable with BT compared to EBRT techniques was substantial, which may be a consideration when choosing a treatment option. Swetha et al. [10] used CT scans of 10 cervical cancer patients treated with HDR BT and made additional IMRT plans for the dosimetric comparison. They found the lowest integral doses of OARs (bladder and rectum) and non-target normal tissue (body minus PTV) in the BT plans. Our results are consistent with findings obtained from prostate and cervical cancer studies [9, 10]. In our study of breast and head and neck cancer patients, BT also resulted in the lowest integral doses when compared with modern EBRT techniques such as IMRT and VMAT. and stereotactic RT with CK. Significant differences were found between the BT and EBRT techniques for integral doses in normal tissue volumes irradiated by 10%, 5%, and 2% of the PD. Since the fractionation schedule and dose prescription for BT and EBRT may differ in clinical practice, we calculated the biologically effective doses in the competing treatment plans. Therefore, the differences observed in the integral doses can be attributed to differences in techniques and dose prescriptions.

It has been shown that BT can compete dosimetrically with modern EBRT techniques at most treatment sites, especially regarding the dose to OARs [21]. The current work complements these findings and shows that due to lower integral doses to normal tissues and OARs, BT is more favorable than modern EBRT techniques using conventional linear accelerators with IMRT and VMAT techniques. For all volumes investigated (normal tissues, OARs), BT always resulted in a lower integral dose compared to IMRT or VMAT. For normal tissue volumes (NT_V10, NT_V5, NT_V2), the difference was always statistically different. For OARs, the difference was statistically significant for the ipsilateral lung and right parotid and for the left parotid and submandibular glands in H&N patients when the PTVs were identical in BT and VMAT plans. With CyberKnife radiotherapy, the integral doses to salivary glands of H&N patients were significantly lower than those of BT. This is probably due to the large number of non-coplanar IMRT beams used in CK treatment. A lower integral dose to normal tissues or any organ at risk is associated with a lower risk of secondary cancer, and this can be an important consideration when choosing an RT technique for a patient. In this respect, BT also has an advantage over EBRT.

A limitation of the current study is that it focuses only on the comparison of integral doses to normal tissues and organs at risk between BT and EBRT techniques. Future work will include calculating and comparing the probability of normal tissue complications and the risk of secondary cancer.

## Conclusion

For patients with breast and head and neck cancer, interstitial brachytherapy results in a lower integral dose to normal tissues and organs at risk compared to modern external beam radiotherapy techniques using conventional linear accelerators. This result can be taken into consideration when choosing the optimal treatment technique. The clinical implications of the reduced integral dose to normal tissues and organs at risk require further detailed investigation.

## Data Availability

The datasets used in this study are available from the corresponding author upon reasonable request.

## References

[1] Nguyen F, Rubino C, Guerin S et al (2008) Risk of a second malignant neoplasm after cancer in childhood treated with radiotherapy: Correlation with the integral dose restricted to the irradiated fields. Int J Radiat Oncol Biol Phys 70:908–915. 10.1016/j.ijrobp.2007.10.034 18262102 10.1016/j.ijrobp.2007.10.034

[2] Hall EJ, Wuu CS (2003) Radiation-induced second cancers: The impact of 3D-CRT and IMRT. Int J Radiat Oncol Biol Phys 56:83–88. 10.1016/S0360-3016(03)00073-7 12694826 10.1016/s0360-3016(03)00073-7

[3] Holtzman AL, Stahl JM, Zhu S et al (2019) Does the Incidence of Treatment-Related Toxicity Plateau After Radiation Therapy: The Long-Term Impact of Integral Dose in Hodgkin’s Lymphoma Survivors. Advan Radiat Oncol 4:699–705. 10.1016/j.adro.2019.07.010 31673663 10.1016/j.adro.2019.07.010 PMC6817558

[4] D’Souza WR II (2003) Nontumor integral dose variation in conventional radiotherapy treatment planning. Medl Phys 30:2065–2071. 10.1118/1.1591991 10.1118/1.1591991 12945972

[5] Shi C, Penagaricano J, Papanikolau N (2008) Comparison of IMRT treatment plans between linac and Helical Tomotherapy based on integral dose and inhomogeneity index. Med Dosim 33(3):215–221. 10.1016/j.meddos.2007.11.001 18674686 10.1016/j.meddos.2007.11.001

[6] Yang R, Xuy S, Jiang W et al (2009) Integral Dose in Three-dimensional Conformal Radiotherapy, Intensity-modulated Radiotherapy and Helical Tomotherapy. Clin Oncol 21:706–712. 10.1016/j.clon.2009.08.002 10.1016/j.clon.2009.08.002 19713087

[7] Ślosarek K, Osewski W, Grządziel A et al (2014) Integral dose: Comparison between four techniques for prostate radiotherapy. Rep Pract Oncol Radiother 20:99–103. 10.1016/j.rpor.2014.10.010 25859398 10.1016/j.rpor.2014.10.010 PMC4338216

[8] Patel S, Drodge S, Jacques A et al (2015) A comparative planning analysis and integral dose of Volumetric Modulated Arc Therapy, Helical Tomotherapy, and Three-dimensional Conformal Craniospinal Irradiation for pediatric medulloblastoma. J Med Imaging Radiat Sci 46(2):134–140. 10.1016/j.jmir.2014.11.003 31052086 10.1016/j.jmir.2014.11.003

[9] Grzywacz VP, Arden JD, Mankuzhy NP et al (2022) Normal Tissue Integral Dose as a Result of Prostate Radiation Therapy: A Quantitative Comparison Between High-Dose-Rate Brachytherapy and Modern External Beam Radiation Therapy Techniques. Adv Radiat Oncol 8(3):101160. 10.1016/j.adro.2022.101160 36896212 10.1016/j.adro.2022.101160 PMC9991537

[10] Shwetha B, Ravikumar M, Palled SR et al (2011) Dosimetric comparison of high dose rate brachytherapy and intensity-modulated radiation therapy for cervical carcinoma. J Med Phys 36(2):111–116. 10.4103/0971-6203.79687 21731228 10.4103/0971-6203.79687 PMC3119952

[11] Richert J, Baier K, Flentje M (2008) Comparison of 60Cobalt and 192Iridium Sources in High Dose Rate Afterloading Brachytherapy. Strahlenther Onkol 184:187–192. 10.1007/s00066-008-1684-y 18398582 10.1007/s00066-008-1684-y

[12] Yadav S, Singh OP, Choudhary S et al (2021) Estimation and comparison of integral dose to target and organs at risk in three-dimensional computed tomography image-based treatment planning of carcinoma uterine cervix with two high-dose-rate brachytherapy sources: 60Co and 192Ir. J Can Res Ther 17:191–197. 10.4103/jcrt.JCRT_199_19 10.4103/jcrt.JCRT_199_19 33723154

[13] Brekner MC, Imhoff D, Rödel C et al (2024) Stereotactic body radiotherapy with volumetric intensity-modulated arc therapy and flattening filter-free beams: dosimetric considerations. Strahlenther Onkol 200:346–357. 10.1007/s00066-023-02181-8 38092967 10.1007/s00066-023-02181-8 PMC10965745

[14] Major T, Stelczer G, Pesznyák C et al (2017) Multicatheter interstitial brachytherapy versus intensity modulated external beam therapy for accelerated partial breast irradiation: A comparative treatment planning study with respect to dosimetry of organs at risk. Radiother Oncol 22:17–23. 10.1016/j.radonc.2016.08.003 10.1016/j.radonc.2016.08.003 27544819

[15] Akiyama H, Pesznyák Cs BD et al (2018) Image guided high-dose-rate brachytherapy versus volumetric modulated arc therapy for head and neck cancer: A comparative analysis of dosimetry for target volume and organs at risk. Radiol Oncol 52:461–467. 10.2478/raon-2018-0042 30422804 10.2478/raon-2018-0042 PMC6287174

[16] Ferenczi Ö, Major T, Fröhlich G et al (2023) Dosimetric comparison of postoperative interstitial high-dose-rate brachytherapy and modern external beam radiotherapy modalities in tongue and floor of the mouth tumours in terms of doses to critical organs. Radiol Oncol 57:516–523. 10.2478/raon-2023-0050 38038418 10.2478/raon-2023-0050 PMC10690754

[17] Aoyama H, Westerly DC, Mackie TR et al (2006) Integral radiation dose to normal structures with conformal external beam radiation. Int J Radiat Oncol Biol Phys 64:962–967. 10.1016/j.ijrobp.2005.11.005 16458781 10.1016/j.ijrobp.2005.11.005

[18] D’Arienzo M, Masciullo SG, de Sanctis V et al (2012) Integral dose and radiation-induced cecondary malignancies: comparison between stereotactic body radiation therapy and three-dimensional conformal radiotherapy. Int J Environ Res Public Health 9:4223–4240. 10.3390/ijerph9114223 23202843 10.3390/ijerph9114223 PMC3524624

[19] Fox C, Hardcastle N, Lim A et al (2015) Calculating integral dose using data exported from a commercial record and verify system. Australas Phys Eng Sci Med 38(2):283–288. 10.1007/s13246-015-0341-x 25869674 10.1007/s13246-015-0341-x

[20] Roth J, Hünig R, Kurtz J (1986) Specific integral dose: A reconsideration of the integral dose conceptSpecific integral dose: A reconsideration of the integral dose concept. Radiother Oncol 5:215–221. 10.1016/s0167-8140(86)80051-2 3085170 10.1016/s0167-8140(86)80051-2

[21] Major T, Fröhlich G, Ágoston P et al (2022) The value of brachytherapy in the age of advanced external beam radiotherapy: a review of the literature in terms of dosimetry. Strahlenther Onkol 198(2):93–109. 10.1007/s00066-021-01867-1 34724086 10.1007/s00066-021-01867-1 PMC8789711

